# Solid phase extraction of tritiated contaminants from tritium-containing waste oils

**DOI:** 10.1007/s10967-016-4953-8

**Published:** 2016-08-05

**Authors:** Andrzej Olejniczak, Jacek Fall, Katarzyna Olejniczak, Marina V. Gustova, Alexandr G. Shostenko

**Affiliations:** 1 Faculty of Chemistry, Nicolaus Copernicus University, ul. Gagarina 7, 87-100 Toruń, Poland; 2 Flerov Laboratory of Nuclear Reactions, Joint Institute for Nuclear Research, Joliot-Curie 6, Dubna, Russia 141980; 3 Oil and Gas Institute-NRI, ul. Lubicz 25A, 31–503 Kraków, Poland

**Keywords:** Tritium-containing waste oils, Solid phase extraction, Lubricating oil oxidation, Acyclic isoprenoids

## Abstract

A solid phase extraction method for removing polar tritiated contaminants from tritium-containing waste oils has been developed. The composition of the degradation products present in the waste oil was determined. The results indicated that upon exposure to tritium gas, fragment methyl ketones, carboxylic acids, and lactones were the main polar products of the mineral-based oil oxidation. The nonpolar fragmentation products included *n*-alkanes, monomethylalkanes, and acyclic isoprenoids and were analogous to those formed during $\gamma$-irradiation of the oil. Various polar and nonpolar fragmentation products containing an isoprenoid skeleton were found to be formed via an oxidative/radiation scission of long-chain acyclic isoprenoids.

**Electronic supplementary material:**

The online version of this article (doi:10.1007/s10967-016-4953-8) contains supplementary material, which is available to authorized users.

## Introduction

The use of lubricating oils under exposure to tritium, elevated temperatures and oxidizing agents results in the accumulation of tritium in the oils and causes structural changes due to oxidation and radiolysis, thereby deteriorating their tribological performance. Consequently, such oils may require frequent changes, and because the amount of accumulated tritium can reach $10^3$ Ci/kg, they should be considered hazardous waste. Tritium-containing waste oils can be generated in fusion test reactors, heavy water reactors, neutron generators and facilities for tritium production, storage, and handling [1].

A considerable amount of attention has been devoted to assessing the radiological hazards associated with spent tritiated oils [1–7]. From a radiological perspective, tritiated oil wastes are considered to be a mixture of three components: insoluble large tritiated organic molecules, soluble tritiated organic molecules, a part that can be classified as tritium-exchangeable, and tritiated water (HTO) [8]. The relative ratio of these components, roughly estimated as 8:1:1 [8], may vary from sample to sample and depends on factors such as oil age, initial composition, and the type of tritium species (e.g., T$_2$, HTO) that the oil has been in contact with.

At present, two main directions for the safe management of tritiated organic wastes (TOW) are being developed. The first is based on the direct immobilization and/or encapsulation of TOW prior to their final disposal. To date, different matrices, including petroleum-absorbing polymers, carbon-based adsorbents, zeolites, alumina, cement, bitumens, and glasses, have been shown to be effective in solidifying, encapsulating, and immobilizing TOW [9–14].

The second approach is based on decontamination, which in its ideal form should allow for both an easy recovery of tritium and the reduction of the radioactivity of wastes to a safe level. Several techniques for the decontamination of TOW have been reported, including vacuum degassing at room or elevated temperatures [15], air-blowing [16], vacuum distillation [16, 17], acid/base- or Raney-nickel-catalyzed isotopic exchange [17, 18], reaction with metallic sodium [19], direct oxidation of the entire oil [20], and adsorption [12, 16, 21]. Because the contents of both volatile tritiated components and easily exchangeable (labile) tritium in TOW are typically low, the methods based on the removal of these components are rather inefficient. The highest decontamination degree of up to 95 % is achievable for polar adsorbents with the appropriate pore structure. In this case, regeneration of the adsorbent and recovery of the tritium are important aspects related to TOW processing. In the literature, two such methods have been proposed: (i) blowing the adsorbent bed with hot air and subsequent high-temperature catalytic oxidation of the desorbed tritiated species [21], and (ii) a combined desorption/isotopic exchange conducted using concentrated sulfuric acid at elevated temperatures, followed by neutralization of the acid to release tritium as HTO [16]. The first method allows for greater enrichment of the generated HTO, which is important for the process economy.

The process of autoxidation of lubricating oils leads to the formation of a large variety of compounds with a broad distribution of molecular masses, thus causing difficulties in their analysis. For this reason, the oxidative stability of the oils and the extent of the oil degradation are typically estimated based on the changes in the average structural parameters or the physicochemical properties. Product identification and mechanistic studies are rare and are typically performed on model compounds, mainly normal alkanes. As shown in [22], such an approach might not necessarily be relevant to real lubricant base oil oxidation. The presence of ionizing radiation is expected to lead to greater scission of alkyl chains, which contributes to the formation of low-molecular-mass products. Consequently, it becomes easier to identify and quantify the individual components. Such knowledge on the chemical forms of tritium dissolved in the oil is important for developing an optimal method for processing tritium-contaminated oils. Moreover, the data can be used to elucidate the mechanisms of oil degradation and for further studies on the suitable selection of base oils and additives, thus enabling an effective reduction of the waste.

In this study, an attempt was made to apply the technique of solid phase extraction (SPE) to the separation of oxidation products from samples of used tritium-containing oils. As we have shown, this effective and rapid method allows a hydrocarbon oil matrix to be obtained that is free of polar tritiated contaminants, and it enables the easy recovery of such contaminants. The obtained fractions representing oxidation products and the hydrocarbon matrix were subjected to chromatographic and spectroscopic analyses and were measured for tritium radioactivity. This made it possible to identify the main products of the oil’s degradation and to determine the detritiation efficiency. Because the initial oil contained considerable quantities of high-molecular-weight acyclic isoprenoids, the analysis of the oxidative/radiation fragmentation products provided an opportunity to verify whether the oxidation mechanisms that occur in real samples are similar to those proposed for branched model compounds. Furthermore, we sought to assess the quality of the regenerated oil via high-temperature simulated distillation.

## Experimental

### Tritium-containing waste oil samples

The samples of tritium-containing waste oils with a specific radioactivity in the range of 10^10^–10^11^ Bq/g [21] were obtained from a tritium-processing facility and were non-aromatic vacuum pump oils of VM-5 type.

### Solid phase extraction (SPE)

The separation of the oxidation products from tritium-containing waste oils was conducted using normal-phase Discovery SPE cartridges (DSC-Si, DSC-Diol, DSC-CN, and DSC-NH$_2$) obtained from Supelco. Prior to fractionation, the cartridges were conditioned with 6 ml of *n*-hexane. A standard mixture consisting of *n*-octane, *n*-decane, *n*-tetradecane, caproic acid, 2-decanone, 4-dodecanolide, 5-decanolide, 5-dodecanolide, and 5-tetradecanolide dissolved in *n*-hexane was used to develop a separation method. The elution was performed by washing the columns with 1.5-ml portions of solvents of increasing eluotropic strength, i.e., *n*-hexane, dichloromethane-methanol (DCM:MeOH) 8:2 v/v, DCM:MeOH 6:4 v/v, and pure methanol. The collected fractions were spiked with an internal standard solution (cyclohexanone in *n*-hexane) and analyzed via GC-FID.

The developed method was used to separate tritiated contaminants from tritium-containing waste oils. Briefly, an oil sample of approximately 0.1 g was dissolved in 0.5 ml of *n*-hexane and loaded onto a SPE cartridge. Nonpolar fractions were eluted with 3 ml of *n*-hexane and were designated as FR1. The polar fractions (FR2) were obtained by eluting the columns with 3 ml of a DCM:MeOH (7:3 v/v) mixture. The fractions were spiked with internal standards: cyclohexanone in *n*-hexane for FR1, and *n*-decane in *n*-hexane for FR2. Then, the solutions were analyzed via GC-MS and GC-FID.

### Gas chromatographic analysis

The compounds present in the obtained fractions were identified by GC-MS analysis performed using an Agilent 6890N gas chromatograph equipped with a 5973 Mass Selective Detector and a 30 m long, 0.25 mm i.d., 0.25 mm thick film HP5-5MS column (Agilent Technologies).

Quantitative analyses were conducted using a Shimadzu GC 17A gas chromatograph equipped with a flame ionization detector (FID) and a 30 m long, 0.25 mm i.d., 0.25 mm thick film ZB-1 column (Zebron). The injector (splitless, time = 0.80 min) and detector temperatures were 360 and 370 °C, respectively. Helium was used as the carrier gas. The initial column temperature was held for 2 min at 40 °C, and then it was increased at 6 °C/min to a final temperature of 350 °C, which was held for 10 min.

A standard mixture of *n*-alkanes (C$_7$–C$_9$ pure compounds and C$_{10}$–C$_{44}$ ASTM D5307 crude oil quantitative standard from Supelco) dissolved in *n*-hexane and spiked with an internal standard (cyclohexanone, 99.9 %) was used to calibrate the detector response to the paraffinic hydrocarbons. The carboxylic acids were analyzed as their methyl esters, which were prepared as described in the Supplementary Information. The calibration for the carboxylic acid methyl esters was performed using a 37-component (C$_{4:0}$–C$_{24:0}$) FAME standard mixture (Supelco). The detector responses to the methyl ketones were calculated relative to those of *n*-alkanes with the most similar boiling points, using the concept of effective carbon number (ECN) [23, 24]. The average value of the dECN correcting factor of −0.9 was obtained based on the analysis of two model compounds (2-octanone and 2-decanone) and was in good agreement with the literature data [23, 24].

### High-temperature simulated distillation (SIMDIS)

High-temperature simulated distillation gas chromatography analysis was used to determine the boiling point distribution of the regenerated oil. The sample was prepared as follows. The nonpolar fraction isolated from used tritium-containing VM-5 oil was concentrated by evaporation under a gentle stream of nitrogen. The obtained residue was dissolved in carbon disulfide to a final concentration of 2 wt% and analyzed using a HT-750 analyzer. The analytical conditions were described elsewhere [25].

### Liquid scintillation counting

To reduce quenching, the SPE procedure for sample preparation was modified by replacing the DCM-MeOH eluent with a toluene-MeOH mixture that has a similar eluotropic strength. Each fraction was spiked with 0.23 ml of *n*-hexane and 2 ml of scintillation cocktail and measured using a Wallac 1409 liquid scintillation counter using a counting time of 300 s. The procedure was repeated three times to confirm reproducibility. The counting efficiency was determined using reference solutions, which were prepared by SPE in the same manner except that (i) the cartridges were loaded with equivalent amounts of pure *n*-hexane rather than tritiated oil sample solution, and (ii) the obtained fractions were spiked with 0.23 ml of the tritium standard in *n*-hexane.

### Infrared spectroscopy (IR)

IR spectra of tritium-containing oils and solvent-free nonpolar fractions were recorded on a Specord M80 spectrometer in the region of 400–4000 cm$^{-1}$ using a KBr cell with a 0.70-mm optical path length.

### X-ray fluorescence (XRF)

The content of metallic impurities in new and used oils was determined by X-ray fluorescence spectrometry using a Canberra spectrometer equipped with a Si(Li) detector with a resolution of 145 eV, as measured for the 5.9 keV line of $^{55}$Fe. A standard ring-shaped radioisotope source of $^{109}$Cd (*E* = 22.16 keV,* T* $_{1/2}$ = 453 days) was used for excitation of X-ray radiation. The measurement time was 30 min. Spectral data were analyzed using the WinAxil software. The concentrations of the elements were calculated using calibration curves obtained for standard solutions of known concentrations.

## Results and discussion

### Characterization of used oils

**Fig. 1:**
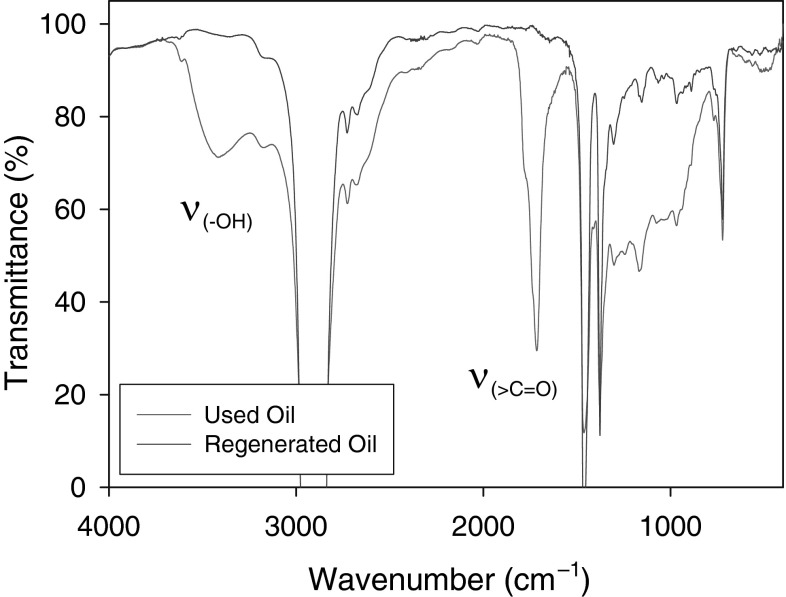
IR spectra of the used tritium-containing VM-5 oil and the regenerated oil

The IR spectrum shown in Fig. 1 indicates that the used VM-5 oil contains significant amounts of oxidation products. The broad band with a maximum at 3400 cm$^{-1}$ confirms the presence of a hydroxyl group, whereas the complex peak at 1715 cm$^{-1}$ is a characteristic of carbonyl group vibrations.

According to [15], tritium-containing waste oils can be contaminated with mercury. Thus, the processing of the oil often involves the removal of not only tritium but also mercury. The XRF analyses showed that in all of our samples, the mercury content was below the detection limit (12 $\upmu$g/g [26]). Only one sample, stored in a metal container for a period of ca. 4 years, contained considerable amounts of metallic impurities, mainly iron (1.44 ± 0.01 %), zinc (0.010 ± 0.001 %), and copper (0.030 ± 0.001 %). Metallic pollutants were not detected in any other samples.

### SPE method development

To develop a procedure for separating the polar oxidation products from used hydrocarbon oils, 4 different SPE phases and a set of eluents of increasing polarity were examined. Optimization was performed using a mixture of model components, i.e., *n*-alkanes, methyl ketones, lactones, and carboxylic acids, all of which represent the classes of compounds identified in the used VM-5 oil.

All of the tested SPE sorbents showed high capability for separating *n*-alkanes from the polar test components. Alkanes were quantitatively eluted with only 2 $\times$ 1.5 ml of *n*-hexane. The application of an additional portion (1.5 ml) of the eluent did not increase the recovery of the test compounds by more than 1 %. GC-FID analyses reveled that for all SPE phases, the obtained *n*-hexane fractions were free from polar test components. The total recoveries of *n*-alkanes obtained for the eluent volume of 4.5 ml are listed in Table 1.

**Table 1:** Total recoveries of *n*-alkanes determined for the various SPE phases using the standard test mixture

Column	Recovery (%)		
	*n*-oktane	*n*-dekane	*n*-tetradecane
DSC-Si	100.7 ± 1.1	100.0 ± 1.0	96.3 ± 0.7
DSC-NH$_2$	99.3 ± 1.3	99.0 ± 1.2	95.0 ± 2.2
DSC-CN	98.0 ± 0.9	97.8 ± 0.8	96.4 ± 2.5
DSC-Diol	100.3 ± 0.6	100.1 ± 2.1	98.4 ± 5.1

The total recoveries of the polar components that eluted from the SPE columns with 3 $\times$ 1.5 ml DCM:MeOH (8:2 v/v) mixture are collected in Table 2. For DSC-Si, DSC-CN, and DSC-Diol columns, quantitative recovery of the polar test components was achieved with a 2 $\times$ 1.5 ml DCM-MeOH mixture. In the case of the aminopropyl phase (DSC-NH$_2$), the fractions eluted by the first two aliquots of 1.5 ml of DCM-MeOH contained only small quantities of ketones and lactones, and virtually no caproic acid. An additional dose (1.5 ml) of DCM-MeOH eluent did not result in an increased recovery for caproic acid but did improve the recovery of ketones and lactones (Table 2). However, the total recoveries of ketones and lactones obtained for DSC-NH$_2$ were still lower than those achieved for the three other SPE sorbents. Such behavior suggests that the interaction of polar test components with the aminopropyl phase is considerably stronger than that with the other sorbents tested. The observed irreversible sorption of carboxylic acids under the applied separation conditions is in accordance with other studies [27]. The washing of carboxylic acids out of the aminopropyl column is possible, but it requires a strong acid, typically formic or acetic, to be applied as an eluent.

**Table 2:** Total recoveries of polar compounds determined for the various SPE phases using the standard test mixture

Column	Recovery (%)					
	Caproic acid	2-decanone	4-dodecanolide	5-decanolide	5-dodecanolide	5-tetradecanolide
DSC-Si	96.3 ± 5.1	94.0 ± 1.1	99.3 ± 10.0	104.0 ± 5.3	100.2 ± 13.0	91.3 ± 14.4
DSC-NH$_2$	0.0 ± 0.1	79.2 ± 2.4	79.3 ± 9.5	82.5 ± 7.0	76.8 ± 7.8	71.7 ± 9.7
DSC-CN	92.9 ± 3.3	89.9 ± 2.7	99.1 ± 12.3	94.9 ± 8.5	101.1 ± 12.0	104.4 ± 6.2
DSC-Diol	86.9 ± 4.4	90.1 ± 2.7	94.2 ± 14.3	100.3 ± 16.4	97.4 ± 14.0	94.3 ± 15.9

### Separation of oxidation products from waste oil

The developed method was applied for the separation of polar oxidation products from used tritium-contaminated VM-5 oil. Following the extraction on SPE columns, two well-separated fractions were obtained: a nonpolar fraction (hydrocarbon oil matrix) and a polar fraction (oil oxidation products). The absence of oxidation products in the regenerated oil, obtained by evaporating the solvent from the nonpolar fraction, was confirmed by IR analysis (Fig. 1).

**Fig. 2:**
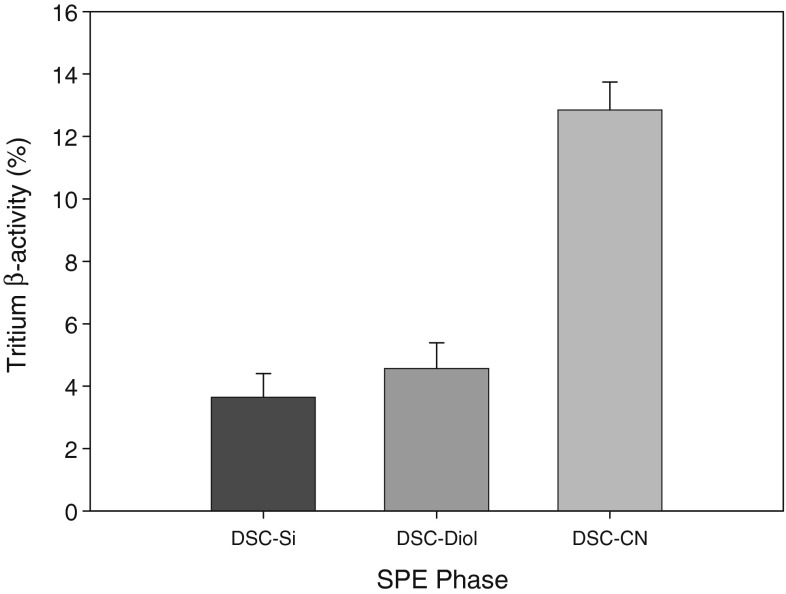
The residual $\beta$-activity of the tritium (%) in the nonpolar fractions that were isolated using different SPE phases

The percentage contributions of tritium activity in the isolated fractions, determined via LSC, are shown in Figure 2. The highest efficiency for tritium removal was achieved for DSC-Si and DSC-Diol phases, which made it possible to obtain nonpolar fractions with tritium activities lower than 5 % of the initial value. The obtained efficiency was similar to or greater than those previously reported for other adsorption-based procedures. For example, stirring undiluted oil with Florisil sorbent for 24 h allowed the tritium content in the oil to be decreased to 8 % [12]. The application of wide-pore adsorbents, such as silica gel, alumina, and zeolites (NaX, CaX, and NaY), allowed the tritium content in the oils to be reduced to 5 % [16, 21]. Adsorption on narrow-pore zeolites (NaA, KA, and NaM) and microporous active carbons was not as effective, allowing purified oils with tritium contents in the range of 36–17 % to be obtained [16, 21]. Compared to these studies, the present SPE-based method allows the extraction time to be significantly reduced. Furthermore, we found that the polar components can be qualitatively eluted to the polar fraction with as little as 3 ml of DCM-MeOH, with no tritium activity loss in the column. The absence of non-reversible sorption of polar components provides an easy and convenient way to regenerate the sorbent and recover tritium.

The high efficiency of polar adsorbents in the removal of tritiated contaminants suggests that the majority of tritium is bonded to oxidation products. This implies that the helium tritide ion ($^3$HeT$^+$) plays a significant role in the process of tritium incorporation into the oils. This species is formed with nearly 95 % efficiency during $\beta$-decay in T$_2$ molecules [28]. Due to the low proton affinity of helium (177.8 kJ/mol) [29], $^3$HeT$^+$ has exceptionally strong acidic properties and is capable of protonation (tritonation) of all organic compounds. The oxidation products present in the system act as effective triton acceptors (i.e., Brønsted bases), thus leading to the accumulation of tritium in subsequent ion-molecule triton transfer reactions.

For the DSC-CN phase, the respective nonpolar fraction contained higher residual tritium activity (ca. 11 %). The lower efficiency of the DSC-CN phase in tritium removal can possibly be explained by taking the hydrogen bonding (HB) capability into account. DSC-Si and DSC-Diol phases posses both HB donor and HB acceptor sites, whereas in the case of the DSC-CN phase, its HB donor capability is significantly suppressed due to the lack of protons in the cyano group and end-capping of residual silanol groups. This means that most of the polar compounds should show lower retention on the DSC-CN phase. For example, carboxylic acids are capable of forming H-bonds with DSC-Si and DSC-Diol phases of both SP–OH$\cdots$O=C(OH)R and SP–(H)O$\cdots$HO–C(O)R type, whereas with the DSC-CN phase, they can only form SP–C$\equiv$N$\cdots$HO–C(O)R H-bonds (SP denotes stationary phase, and R stands for residual alkyl of the analyte molecule). Ketones and lactones are capable of forming SP–OH$\cdots$O hydrogen bonds with both DSC-Si and DSC-Diol phases, but they are unable to form H-bonds with the DSC-CN phase, and only dipole-dipole interactions are responsible for separation in this case. Note that lower recoveries on the DSC-CN phase were not observed for the test components; however, compounds of relatively low molecular mass were used in that case.

### Characterization of the nonpolar fraction

**Fig. 3:**
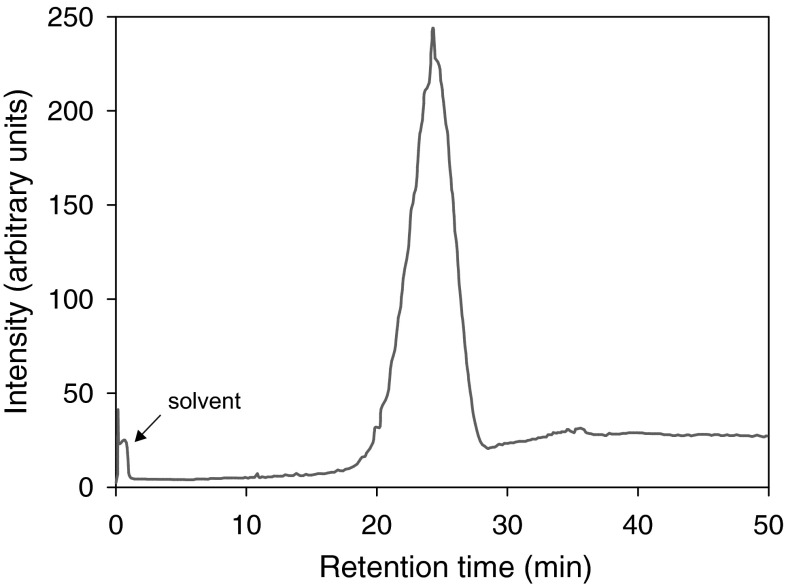
SIMDIS high-temperature gas chromatogram of the regenerated oil

A high-temperature gas chromatogram of the regenerated oil obtained from the nonpolar fraction is shown in Fig. 3. The initial and final boiling points of the regenerated oil, determined via SIMDIS, are 265 and 717 °C, respectively. Such a wide boiling range suggests that both low-molecular-mass products (bp < 480 °C), resulting from the scission of hydrocarbon chains, and high-molecular-mass products (bp > 540 °C) due to cross-linking were formed during service. Both components are expected to be absent in fresh oil, and their presence in vacuum oils is undesirable. The former, due to their high volatility, reduce the performance of equipment, whereas the latter contribute to the increase in the viscosity of the oil and present a potential risk of accumulating in the form of deposits. We found that the concentration of low-molecular-mass products was below 1 wt% but that of high-molecular-mass components was more significant (ca. 14 wt%). It is well known, however, that the products of radiation-induced cross-linking are typically more branched than the parent compounds, and as a result, they typically show relatively low melting points. Thus, the observed changes do not preclude the possibility of re-using regenerated oils.

**Fig. 4:**
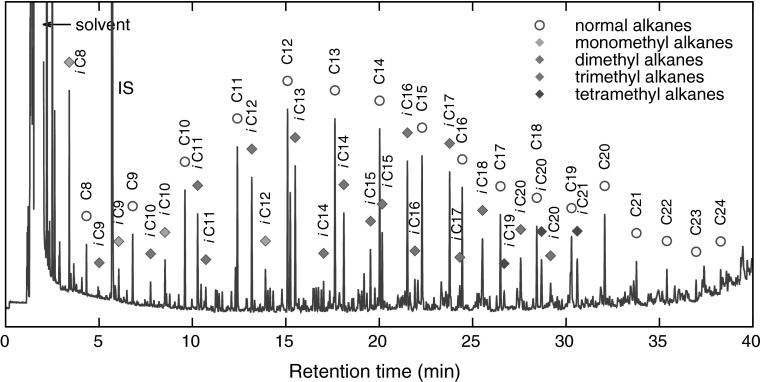
Chromatogram of the low-molecular-mass products present in the nonpolar fraction. Normal and methyl-branched alkanes were designated as ‘C*x*’ and ‘*i*C*x*’, respectively, where *x* denotes the number of carbon atoms (*N*_c_). (Color figure online)

The structural identification of the low-molecular-mass products present in the nonpolar fraction was performed using GC-MS. The obtained chromatogram (Fig. 4) showed the presence of *n*-alkanes, monomethylalkanes, and acyclic isoprenoids (isopranes) ranging from C7–C24 (Table 3).

**Table 3:** The main classes of compounds identified in used tritium-containing VM-5 oil

Compound type	Structure		Designation^a^
Normal alkanes	C$_n$H$_{2n+2}$	*n* = 8 to 24	C8–C24
Monomethyl and isoprenoid alkanes	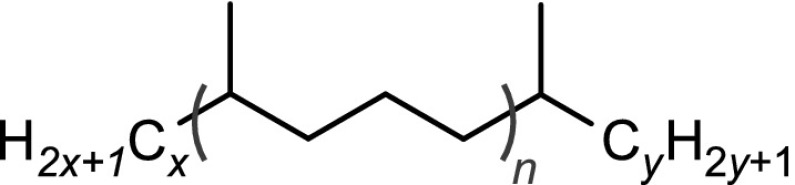	*n* = 0 to 3	*i*C8–*i*C22
Normal methyl ketones	CH$_3$C(O)C$_n$H$_{2n+1}$	*n* = 4 to 20	K6–K22
Isoprenoid ketones	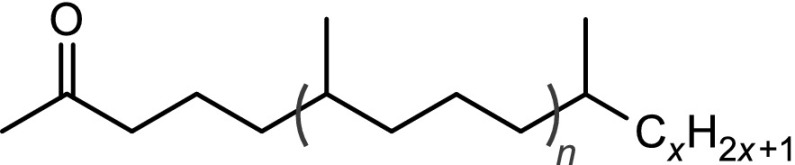	*n* = 0 to 2	*i*K8–*i*K22
Normal $\gamma$-lactones	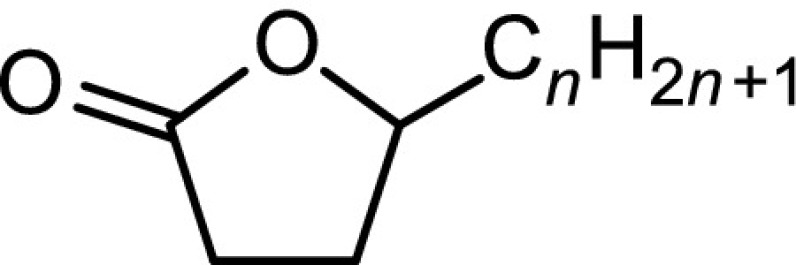	*n* = 1 to 13	L5–L17
Isoprenoid $\gamma$-lactones	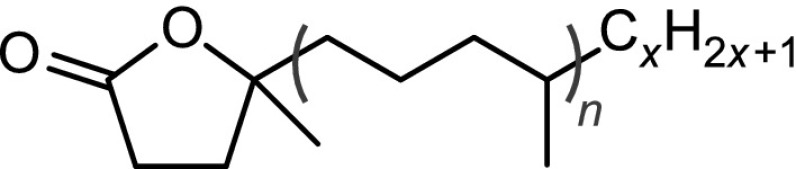	*n* = 0 to 3	MeL5–MeL22
Normal carboxylic acids	C$_n$H$_{2n+1}$COOH	*n* = 3 to 19	A4–A20
Monomethyl and isoprenoid carboxylic acids			
2,6,10,...-Methylbranched	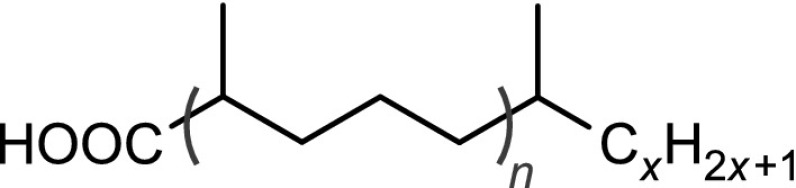	*n* = 0 to 3	$i_2$A5–$i_2$A20
3,7,11,...-Methylbranched	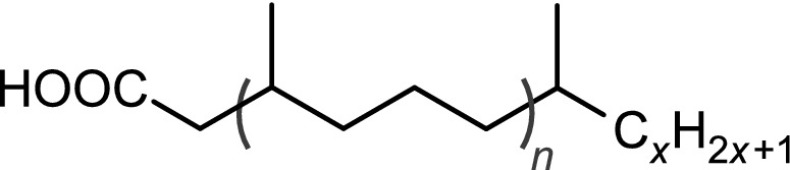	*n* = 0 to 3	$i_3$A5–$i_3$A21
4,8,12,...-Methylbranched	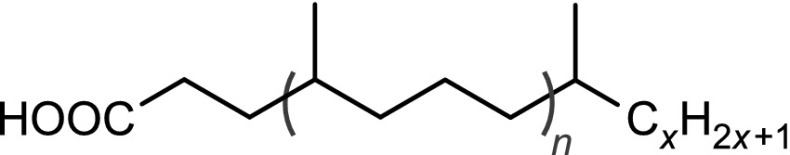	*n* = 0 to 3	$i_4$A6–$i_4$A22

^a^The complete list of identified isoprenoid compounds is available in Tables S1 and S2 in the Sup. Info.

$$x\le y=1,2,3,5$$

**Fig. 5:**
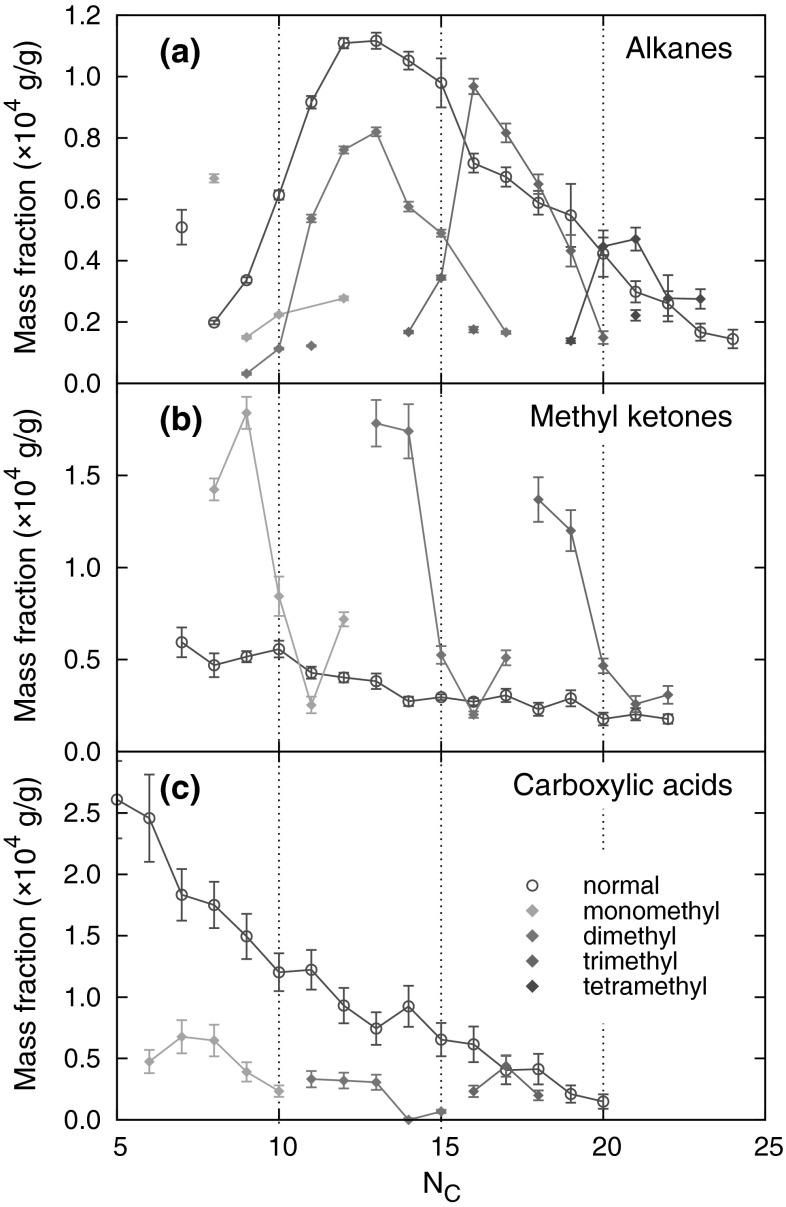
Concentration profiles (mass fraction vs. the number of carbon atoms* N*_c_) of normal and branched compounds: alkanes present in the nonpolar fraction (**a**); ketones (**b**) and fatty acids (**c**) present in the polar fraction. (Color figure online)

In the instances where the interpretation of the mass spectral fragmentation patterns was ambiguous, the identification of the components was confirmed by comparing their Kovats retention indices with those reported in the literature or determined for the products of the $\gamma$-radiolysis of the standard isoprenoids (squalane and phytane). Radiation fragmentation of squalane made it possible to easily obtain a homologous series of methyl-branched alkanes, such as 2,6-dimethyl-, 2,6,10-timethyl-, and 2,6,10,15-tetramethylalkanes [30]. Regarding phytane, it was also possible to obtain 3,7-dimethyl-, 3,7,11-timethyl-, and 3,7,11,15-tetramethylalkanes. The high content of methyl-branched alkanes, relative to that of *n*-alkanes (Fig. 5a), suggests that they were formed as a result of the oxidative and/or radiolytic scission of high-molecular-mass isopranes that were present in the initial oil in high quantities. This assumption was confirmed by $^{13}$C NMR spectroscopy (data not shown) by the presence of intense signals with chemical shifts of 24.45 and 24.79 ppm. These signals are characteristic of C atoms in the –CC(C)C**C**CC(C)C– and CC(C)C**C**CC(C)C– fragments, respectively [31]. The occurrence of the high relative amounts of some methyl-branched fragmentation products and the absence of others can easily be explained, first, by the abstraction of the tertiary hydrogen atoms being favored over the primary and secondary ones, and second, by a chain scission that preferably occurs between the highly substituted carbons. A detailed study on the radiation-induced scission of these isoprenoids will be reported elsewhere.

The same classes of compounds were identified in the VM-5 oil sample that was exposed to $\gamma$-radiation under oxygen-free conditions. However, they show different distributions of concentrations, i.e., the content of low-molecular-weight hydrocarbons (<C$_{12}$) in the $\gamma$-irradiated oil was evidently higher (data not shown). The reduction in the concentration of the volatile hydrocarbons in used oil was most likely a result of the operating conditions (e.g., elevated temperature, reduced pressure), as well as a long storage period.

### Characterization of the polar fraction

**Fig. 6:**
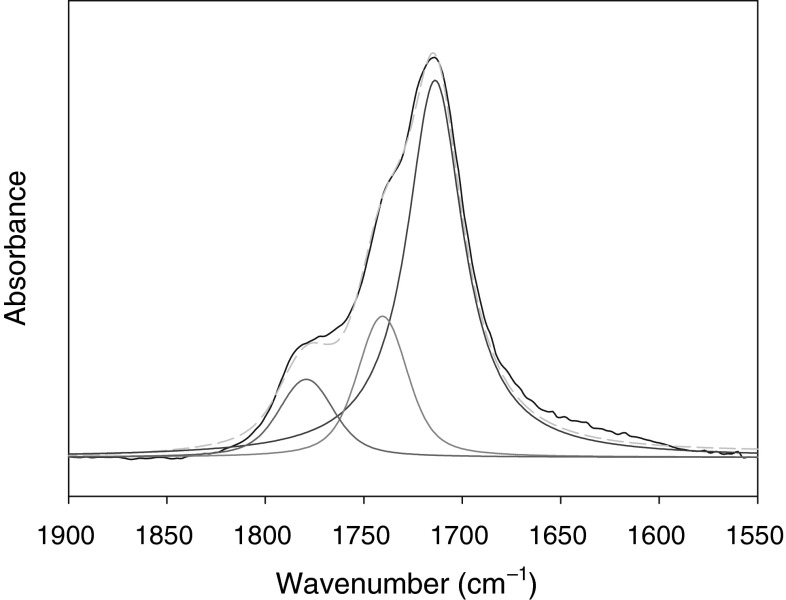
Deconvolution of the carbonyl region of the IR spectrum of used oil

**Fig. 7:**
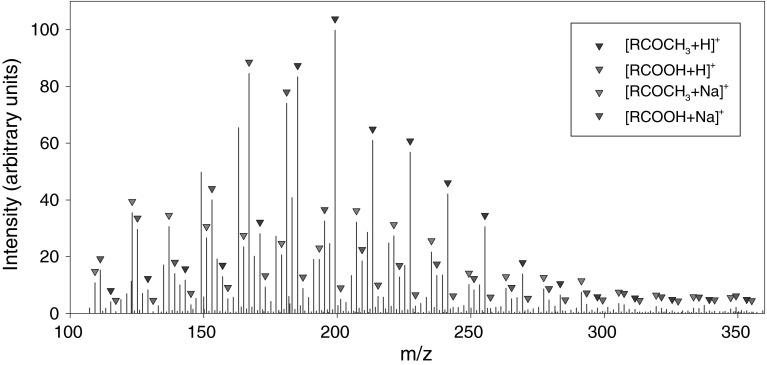
ESI-TOF-MS spectrum of the polar fraction isolated from the used VM-5 oil. (Color figure online)

The portion of the IR spectrum covering the carbonyl region (Fig. 6) is complex, which indicates the presence of a variety of different types of carbonyl carbon compounds in the waste oil. The deconvolution of this band suggests that methyl ketones, carboxylic acids, and lactones were most likely to occur in the polar fraction. A similar conclusion can be drawn from the analysis of the ESI-MS spectrum shown in Fig. 7, which exhibits the presence of a series of peaks in the mass range *m/z* 100–350 due to the protonated molecular ions (M + H$^+$) and sodium adduct ions (M + Na)$^+$. The main classes of compounds identified in the used tritium-containing oil are listed in Table 3.

**Fig. 8:**
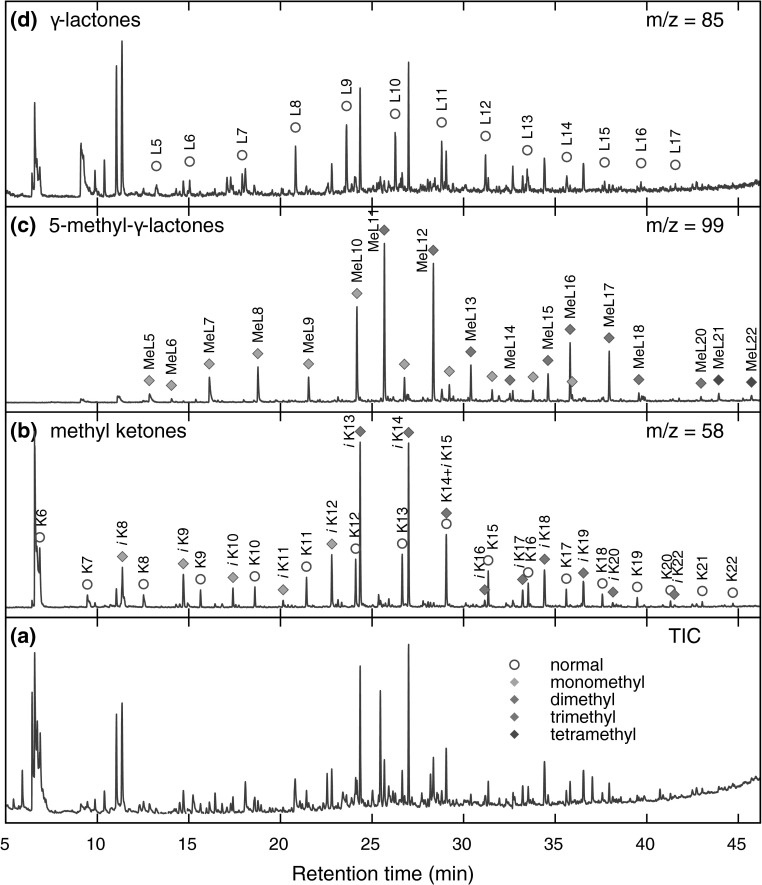
GC-MS analysis of the underivatized compounds present in the polar fraction. For marker description, see Table S1 in the Sup. Info. (Color figure online)

**Fig. 9:**
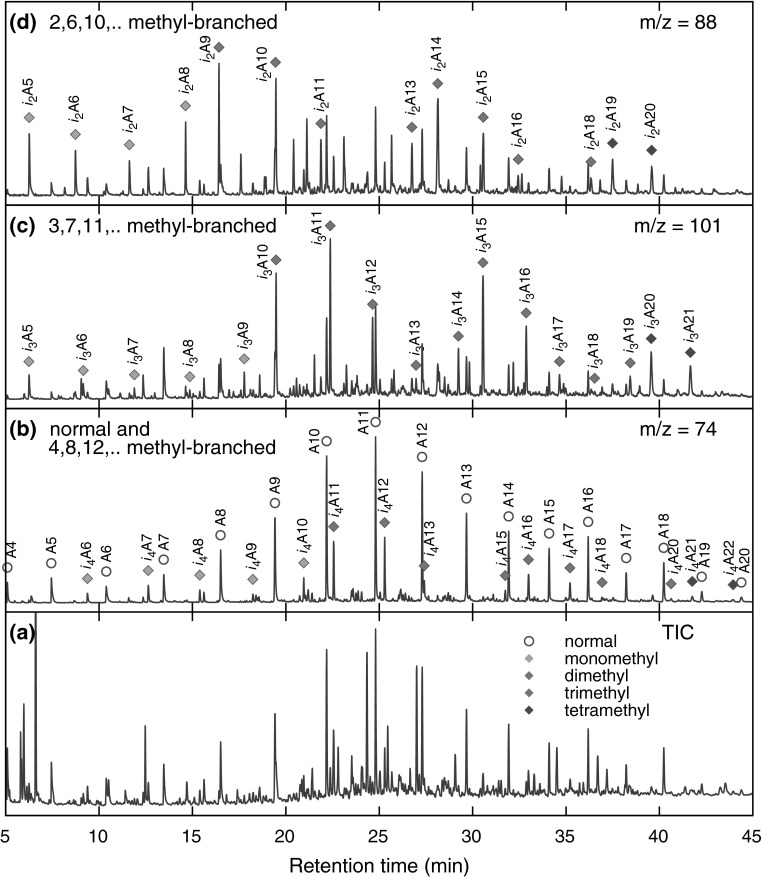
GC-MS analysis of fatty acid methyl esters (derivatized polar fraction). For marker description, see Table S1 in the Sup. Info. (Color figure online)

**Scheme 1:**
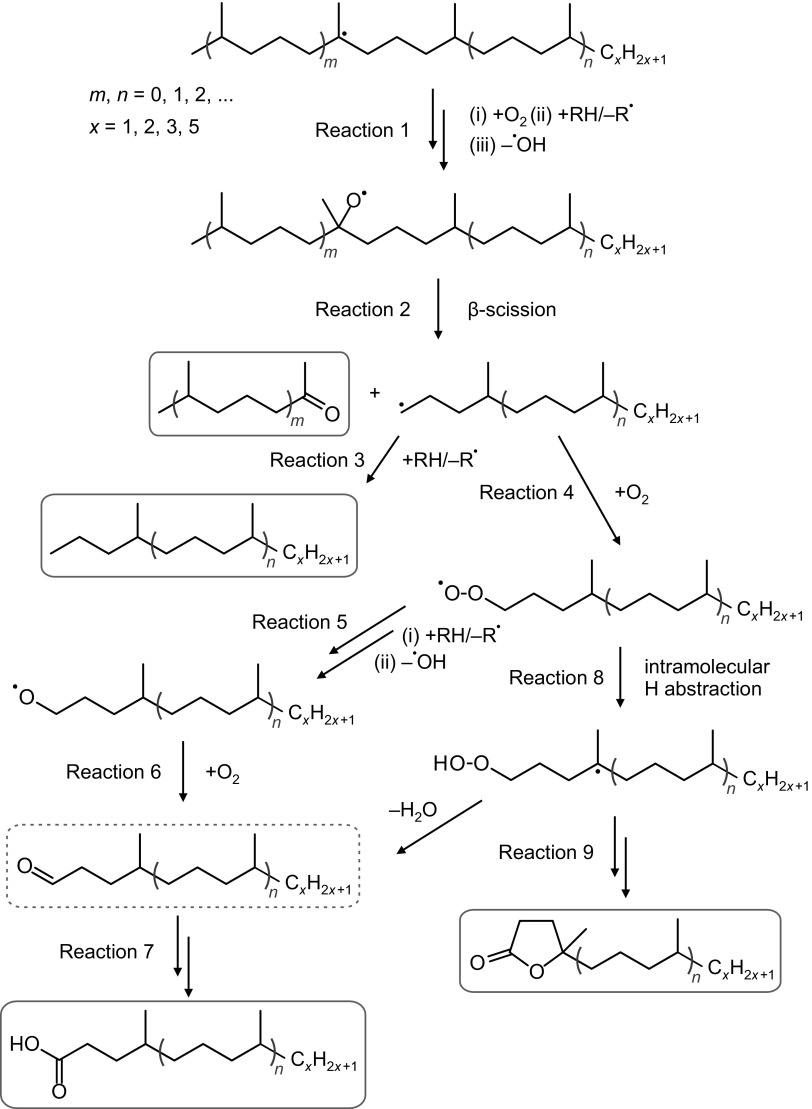
Oxidation reactions of the acyclic isoprenoid alkanes. The identified stable products are marked in * red*. (Color figure online)

To obtain deeper insights into the chemical composition of the polar fraction, we used characteristic fragment ions and retention indices in the GC-MS analysis. Thus, for methyl ketones, the characteristic mass fragments used were *m/z* 43, 58, and M$-18$, resulting from $\alpha$-fragmentation, McLaffery’s rearrangement, and loss of a water molecule, respectively. $\gamma$-Lactones and 5-methyl-$\gamma$-lactones were identified by base peaks at *m/z* 85 and 99, respectively, resulting from the elimination of the main alkyl chain from a lactone ring. Carboxylic acids were analyzed as their respective methyl esters. For straight-chain FAMEs, *m/z* 74 was the base peak and *m/z* 87 was quite intense. Acyclic isoprenoid FAMEs with methyl branches at positions 4, 8, 12, and so forth displayed similar fragmentation patterns, with intense *m/z* 74 and 87 ions; however, in this case, *m/z* 87 was the base signal. In the case of isoprenoid FAMEs with methyl branches at positions 3, 7, 11, and so forth (pseudo-homologues of phytanoic acid), *m/z* 101 was the main signal, and the *m/z* 74 ion was quite prominent. Finally, isoprenoid FAMEs with methyl branches at positions 2, 6, 10, and so forth (pseudo-homologues of pristanoic acid) were recognized using the characteristic ions *m/z* 88 and 101; in this case, *m/z* 88 was the most intense signal, and *m/z* 74 was of low intensity. The complete list of identified isoprenoid compounds, along with their retention times on a HP-5MS column, is compiled in Tables S1 and S2 (Sup. Info.). The respective partial chromatograms for methyl ketones, $\gamma$-lactones, and 5-methyl-$\gamma$-lactones are presented in Fig. 8, and those of FAMEs are shown in Fig. 9. As shown, the partial chromatogram for methyl ketones consists of two series of peaks: the first one originates from the normal methyl ketones, whereas the second one is due to the isoprenoid methyl ketones (Table S1, Sup. Info.; compounds *i*K8–*i*K22). Similarly, as for the nonpolar fragmentation products, the characteristic feature is that some of the isoprenoid ketones, particularly those with main chains consisting of methyl and ethyl termini (compounds *i*K8, *i*K9, *i*K13, *i*K14, *i*K18, and *i*K19 in Table S1, Sup. Info.), are present in higher concentrations than their normal isomers (Fig. 5b). The partial chromatogram for 5-methyl-$\gamma$-lactones mainly consists of peaks representing compounds with isoprenoid alkyl chains. Note that the chromatographic pattern of 5-methyl-$\gamma$-lactones is very similar to that of isoprenoid methyl ketones but is shifted toward higher retention times due to the presence of an additional oxygen atom, which lowers volatility. In contrast, a partial chromatogram for $\gamma$-lactones shows prominent signals only for normal homologues. Most of the above observations can be explained by assuming a mechanism similar to that deduced by Stark et al. for the low-temperature autoxidation of the model branched alkanes [22]. The initial step in this mechanism is the formation of parent alkyl radicals, which in our case can proceed via the abstraction of a H atom by an oxygen-centered radical or by a $\beta$-irradiation-induced C–H bond rupture. Both processes are expected to be quite selective, producing preferentially tertiary alkyl radicals [32–34]. These radicals, in a series of subsequent reactions involving dioxygen addition, H atom abstraction, and dissociation of the resulting hydroperoxide, give rise to the formation of tertiary alkoxyl radicals (Reaction 1, Scheme 1), which in turn might decompose via $\beta$-scission (Reaction 2), resulting in the formation of the fragmentary products: methyl ketone and primary alkyl radical. Scheme 1 is drawn to illustrate the transformations of the isoprenoid alkanes, but similar processes can also occur for monomethyl branched alkanes to create normal methyl ketones and alkyl radicals. Primary alkyl radicals can behave in two ways: they can abstract hydrogen atoms with the formation of fragmentary alkanes (Reaction 3), or they can react with dioxygen, yielding peroxy radicals (Reaction 4), which in a series of reactions (Reactions 5–7) gives either normal or 4, 8, 12, etc. methyl branched carboxylic acids (products $i_4$A6–$i_4$A22, Table S2, Sup. Info.). Further support for this mechanism is provided by the detection in the derivatized sample trace amounts of normal and branched aldehydes (as dimethyl acetals, characteristic ion *m/z* 75), which are assumed to be stable intermediates that are present in low steady state concentrations. Alternative routes for the transformation of the primary isoprenoid peroxy radicals involves an intramolecular H-atom abstraction via a 7-membered intermediate (Reaction 8). The alkyl hydroperoxide radical formed can then add dioxygen and undergo further reactions, thus forming 5-methyl-$\gamma$-lactones (Reaction 9). The formation of normal $\gamma$-lactones is typically assumed to proceed from secondary alkyl radicals via Goosen and Kindermans mechanism [35]. The mechanism, although highly likely for the formation of normal products, would require the absence of branching in the non-leaving moiety. Thus, it cannot be precluded that normal $\gamma$-lactones are formed in a similar way that 5-methyl-$\gamma$-lactones are, but from monomethyl branched structures rather than isoprenoid ones. In this work, we were unable to detect high-molecular-weight oxidation products, including, for example, alcohols formed from parent alkoxy radicals. Such components, however, might undergo discrimination on a chromatographic column. Note that the formation of some of the minor products identified cannot be explained by assuming only C–H bond scission. For example, the creation of the pseudo-homologues of pristanoic and phytanoic acids (products $i_2$A5–$i_2$A20 and $i_3$A5–$i_3$A21, Table S2, Sup. Info.) would require the breaking of C–C bonds between the secondary carbons of the isoprenoid chain. Such chain scission can be caused by $\beta$-irradiation and should produce pseudo-homologues of both acids in comparable quantities, which was indeed observed. Such a process is also believed to contribute to isoalkane fragmentation products.

## Conclusions

A quick and simple procedure was developed by using the SPE technique for the separation of the oxidation products from the waste tritium-contaminated oils. Due to the high efficiency of the removal of the polar tritiated compounds, the normal-phase sorbents can be applied during one of the initial stages of the waste oil decontamination/rerafination, allowing for the removal of up to 95 % of tritium. The application of SIMDIS enables the assessment of the quality of the regenerated oil that was obtained from the nonpolar fraction.

The main products of the mineral-based oil oxidation under exposure to tritium gas includes fragment methyl ketones, carboxylic acids, and lactones. In accordance with the literature [16, 21], these products were found to accumulate most of the tritium. The changes in the nonpolar fraction are manifested by the occurrence of both low-molecular-mass and high-molecular-mass products, which can potentially be responsible for the deterioration of the oil’s properties. The identified low-molecular-mass compounds (*n*-alkanes, monomethylalkanes, and acyclic isoprenoids) are analogous to those that appear during $\gamma$-irradiation of the VM-5 oil under oxygen-free conditions. The oxidative/radiation-induced scission of the long-chain acyclic isoprenoids that are present in the initial oil leads to a variety of fragmentation products with an isoprenoid skeleton, thus confirming, on a real sample, the significance of the competing reaction pathways that were previously deduced for model branched alkanes.

## Electronic supplementary material

Below is the link to the electronic supplementary material.

Supplementary material 1 (PDF 25 kb)
