# Supplementary material for: Solid phase extraction of tritiated contaminants from tritium-containing waste oils
Source: J Radioanal Nucl Chem. 2016 Aug 5;310(3):1085–97. doi: 10.1007/s10967-016-4953-8 (PMC5110708; doi:10.1007/s10967-016-4953-8)
Supplement: Supplementary file 1 — Supplementary material 1 (PDF 25 kb) [file 10967_2016_4953_MOESM1_ESM.pdf]

Supplementary material for: Solid Phase Extraction of Tritiated Contaminants from Tritium-containing Waste Oils

Andrzej Olejniczak,[*,†,‡] Jacek Fall,[¶] Katarzyna Olejniczak,[†,‡] Marina V. Gustova,[‡] and Alexandr G. Shostenko[†]

Faculty of Chemistry, Nicolaus Copernicus University, ul. Gagarina 7, 87-100 Toruń, Poland. Flerov Laboratory of Nuclear Reactions, Joint Institute for Nuclear Research, Dubna, 141980, Russia.

E-mail: aolejnic@chem.umk.pl

Preparation of fatty acid methyl esters (FAMEs)

The fatty acids present in FR2 that were quantified as their methyl esters were prepared by treating the fraction with 2 ml of 10% (v/v) sulfuric acid in methanol. The esterification reaction was performed in a sealed ampule at 60 °C for 30 min under vigorous shaking. After the mixture was allowed to cool to room temperature, the excess acid was neutralized with saturated aqueous sodium bicarbonate. Then, the sample was extracted 4 times with 1 ml of *n*-hexane. The organic layers were combined, dried with 1 g of anhydrous sodium sulfate for 30 min, and filtered into a 10-ml volumetric flask. The remaining drying agent was rinsed with an additional 1 ml of *n*-hexane, and the extracts were combined, spiked with an internal standard, and submitted for GC-MS and GC-FID analyses.

Table S1: Low-molecular-mass products found in the polar fraction

Peak no.	Compound name	Structure	Retention time (min)
iK8	6-methyl-2-heptanone	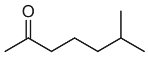	11.37
iK9	6-methyl-2-octanone	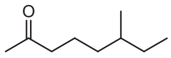	14.70
iK10	6-methyl-2-nonanone	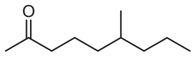	17.41

Continued on next page

[*]To whom correspondence should be addressed
[†]Nicolaus Copernicus University
[‡]Joint Institute for Nuclear Research
[¶]Previous address: Institute of Petroleum Processing, ul. Łukasiewicza 1, 31-429 Kraków, Poland

Table S1 – *Continued from previous page*

Peak no.	Compound name	Structure	Retention time (min)
iK12	6-methyl-2-undecanone	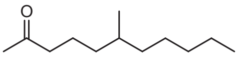	22.81
iK13	6,10-dimethyl-2-undecanone	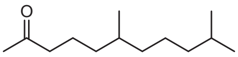	24.36
iK14	6,10-dimethyl-2-dodecanone	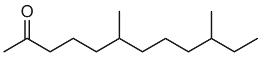	27.00
iK15	6,10-dimethyl-2-tridecanone	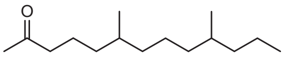	29.06
iK17	6,10-dimethyl-2-pentadecanone	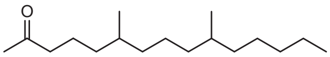	33.22
iK18	6,10,14-trimethyl-2-pentadecanone	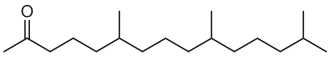	34.42
iK19	6,10,14-trimethyl-2-hexadecanone	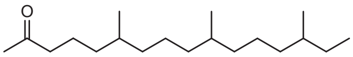	36.54
iK20	6,10,14-trimethyl-2-heptadecanone	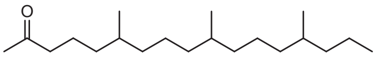	38.16
iK22	6,10,14-trimethyl-2-nonadecanone	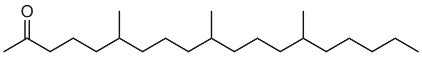	41.50
MeL6	4-methylpentan-4-olide	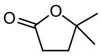	14.04
MeL7	4-methylhexan-4-olide	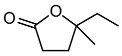	16.12
MeL8	4-methylheptan-4-olide	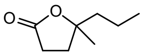	18.77
MeL9	4-methyloctan-4-olide	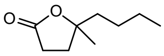	21.54
MeL10	4-methylnonan-4-olide	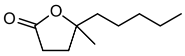	24.18
MeL11	4,8-dimethylnonan-4-olide	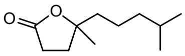	25.67
MeL12	4,8-dimethyldecan-4-olide	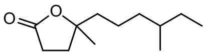	28.34
MeL13	4,8-dimethylundecan-4-olide	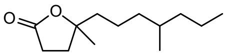	30.39
MeL14	4,8-dimethyldodecan-4-olide	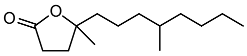	32.53
MeL15	4,8-dimethyltridecan-4-olide	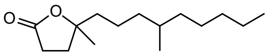	34.61
MeL16	4,8,12-trimethyltridecan-4-olide	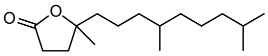	35.82
MeL17	4,8,12-trimethyltetradecan-4-olide	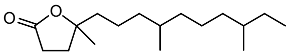	37.95
MeL18	4,8,12-trimethylpentadecan-4-olide	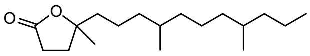	39.57
MeL20	4,8,12-trimethylheptadecan-4-olide	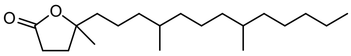	42.96
MeL21	4,8,12,16-tetramethylheptadecan-4-olide	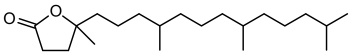	43.92
MeL22	4,8,12,16-tetramethyloctadecan-4-olide	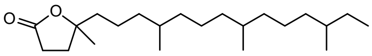	45.72

Table S2: Fatty acids methyl esters found in the derivatized polar fraction

Peak no.	Compound name	Structure	Retention time (min)
i_2A5	methyl 2-methylbutanoate	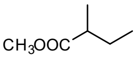	6.26
i_2A6	methyl 2-methylpentanoate	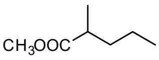	8.74
i_2A7	methyl 2-methylhexanoate	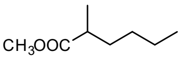	11.63
i_2A8	methyl 2-methylheptanoate	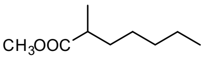	14.63
i_2A9	methyl 2,6-dimethylheptanoate	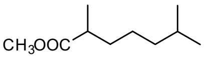	16.42
i_2A10	methyl 2,6-dimethyloctanoate	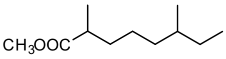	19.45
i_2A11	methyl 2,6-dimethylnonanoate	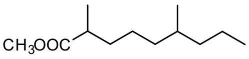	21.88
i_2A13	methyl 2,6-dimethylundecanoate	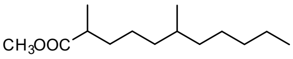	26.75
i_2A14	methyl 2,6,10-trimethylundecanoate	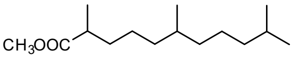	28.13
i_2A15	methyl 2,6,10-trimethyldodecanoate	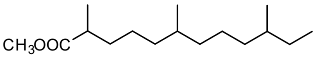	30.57
i_2A16	methyl 2,6,10-trimethyltridecanoate	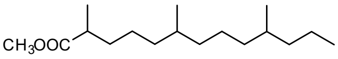	32.44
i_2A18	methyl 2,6,10-trimethylpentadecanoate	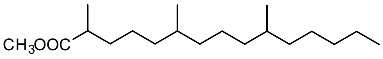	36.33
i_2A19	methyl 2,6,10,14-tetramethylpentadecanoate	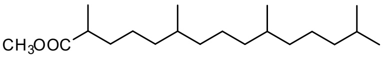	37.49
i_2A20	methyl 2,6,10,14-tetramethylhexadecanoate	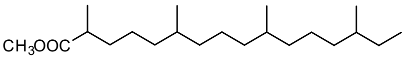	39.58
i_3A5	methyl 3-methylbutanoate	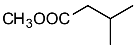	6.25
i_3A6	methyl 3-methylpentanoate	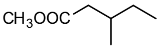	9.15
i_3A7	methyl 3-methylhexanoate	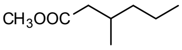	11.89
i_3A8	methyl 3-methylheptanoate	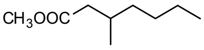	14.86
i_3A9	methyl 3-methyloctanoate	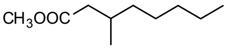	17.77
i_3A10	methyl 3,7-dimethyloctanoate	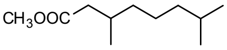	19.48
i_3A11	methyl 3,7-dimethylnonanoate	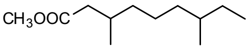	22.38
i_3A12	methyl 3,7-dimethyldecanoate	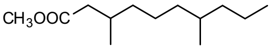	24.64
i_3A13	methyl 3,7-dimethylundecanoate	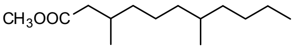	26.97
i_3A14	methyl 3,7-dimethyldodecanoate	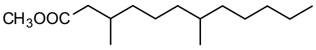	29.24
i_3A15	methyl 3,7,11-trimethyldodecanoate	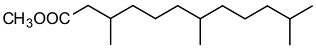	30.55
i_3A16	methyl 3,7,11-trimethyltridecanoate	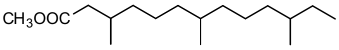	32.87
i_3A17	methyl 3,7,11-trimethyltetradecanoate	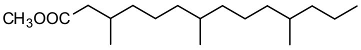	34.65

Continued on next page

Table S2 – *Continued from previous page*

Peak no.	Compound name	Structure	Retention time (min)
i_3A18	methyl 3,7,11-trimethylpentadecanoate	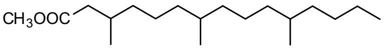	36.53
i_3A19	methyl 3,7,11-trimethylhexadecanoate	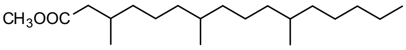	38.43
i_3A20	methyl 3,7,11,15-tetramethylhexadecanoate	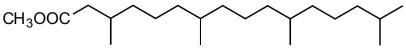	39.56
i_3A21	methyl 3,7,11,15-tetramethylheptadecanoate	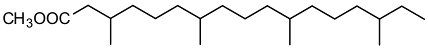	41.66
i_4A6	methyl 4-methylpentanoate	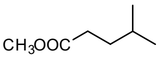	9.38
i_4A7	methyl 4-methylhexanoate	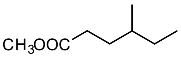	12.63
i_4A8	methyl 4-methylheptanoate	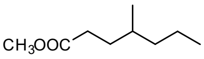	15.39
i_4A9	methyl 4-methyloctanoate	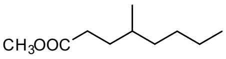	18.24
i_4A10	methyl 4-methyloctanoate	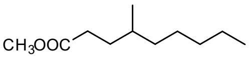	20.96
i_4A11	methyl 4,8-dimethylnonanoate	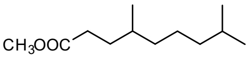	22.56
i_4A12	methyl 4,8-dimethyldecanoate	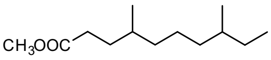	25.29
i_4A13	methyl 4,8-dimethylundecanoate	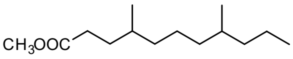	27.41
i_4A15	methyl 4,8-dimethyldodecanoate	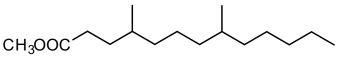	31.74
i_4A16	methyl 4,8,12-trimethyltridecanoate	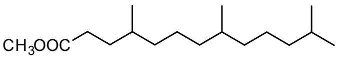	32.99
i_4A17	methyl 4,8,12-trimethyltetradecanoate	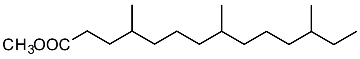	35.21
i_4A18	methyl 4,8,12-trimethylpentadecanoate	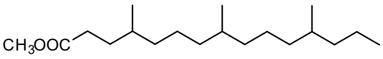	36.93
i_4A20	methyl 4,8,12-trimethylhexadecanoate	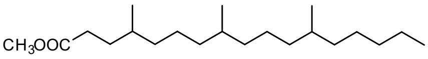	40.63
i_4A21	methyl 4,8,12,16-tetramethylheptadecanoate	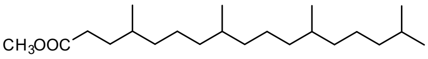	41.76
i_4A22	methyl 4,8,12,16-tetramethyloctadecanoate	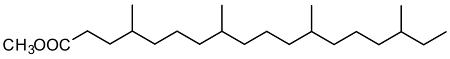	43.95
